# Finite Element Analysis of the Load-Bearing Components of a Stackable Surgical Guide System During Flapless Implant Osteotomy in a Fully Edentulous Maxilla

**DOI:** 10.3390/ma19194103

**Published:** 2026-09-25

**Authors:** Ionut Gabriel Ghionea, Corina Marilena Cristache, Stefan Cristian Dumitru, Cristian Ioan Tarba, Oana Elena Burlacu Vatamanu, Irina Adriana Beuran

**Affiliations:** 1 Manufacturing Engineering Department, Faculty of Industrial Engineering and Robotics, National University of Science and Technology Politehnica Bucharest, 313 Splaiul Independentei, 060042 Bucharest, Romania; gabriel.ghionea@upb.ro (I.G.G.); cristian.tarba@upb.ro (C.I.T.); 2 Department of Dental Techniques, “Carol Davila” University of Medicine and Pharmacy, 8, Eroii Sanitari Blvd., 050474 Bucharest, Romania; irina.beuran@umfcd.ro; 3 Smart Biomaterials and Applications Master Program, Faculty of Medical Engineering, National University of Science and Technology POLITEHNICA Bucharest, 1-7 Gh Polizu Street, 011061 Bucharest, Romania; stefan.dumitru2202@gmail.com; 4 Doctoral School, “Carol Davila” University of Medicine and Pharmacy, 37 Dionisie Lupu Street, 020021 Bucharest, Romania; oana-elena.burlacu-vatamanu@drd.umfcd.ro

**Keywords:** finite element analysis, stackable surgical guide, guided implant surgery, fully edentulous maxilla, flapless implant placement, dental biomaterials, methacrylate-based photopolymer resin, titanium fixation pins

## Abstract

**Highlights:**

- Finite element evaluation of a stackable surgical guide assembly for a fully eden-tulous maxilla.
- Under the maximum simulated drilling load, calculated stresses remained below the selected reference strengths in both contact scenarios.
- The polymeric guides exhibited substantially greater relative material utilization than the titanium fixation pins.
- Maximum predicted guide deformation was 0.093 mm, indicating limited defor-mation under the selected simulated loading conditions.
- Enhanced printable resin formulations may represent a relevant direction for fu-ture mechanical optimization of the guide system.

**Abstract:**

Stackable surgical templates enable flapless, prosthetically driven implant placement and immediate provisionalization in full-arch rehabilitation; however, intraoperative guide fracture remains a recognized complication, and the biomechanical behavior of the multi-component assembly on the edentulous maxilla has not been fully characterized. This in silico study used 3D modeling, surface refinement and finite element analysis (CATIA V5) to evaluate stress and displacement distribution in a model comprising the maxilla, polymeric base guide, implant osteotomy guide, and five grade-4 titanium fixation pins, derived from a prosthetically driven virtual plan. Methods: Materials were modeled as homogeneous, isotropic, and linearly elastic. A maximum simulated axial drilling force of 10.14 N and an associated equivalent torsional load derived from the calculated cutting torque were applied for a 2 mm diameter osteotomy under Scenario 1 (idealized no-sliding contact) and Scenario 2 (frictional contact). Results: Under Scenario 1, the maximum von Mises stresses were 2.34 MPa in the Maxilla, 3.18 MPa in the Base guide, 4.32 MPa in the Implant osteotomy guide, and 1.13–3.26 MPa in the Fixation pins. The highest polymer stress occurred in the Implant osteotomy guide (4.32 MPa; 7.2% of the 60 MPa reference strength). Under Scenario 2, the maximum stresses were 2.48 MPa in the Maxilla, 7.63 MPa in the Base guide, 2.38 MPa in the Implant osteotomy guide, and 1.11–3.94 MPa in the Fixation pins, with the Base guide showing the highest polymer utilization (7.63 MPa; 12.7%). Across both contact formulations, the polymeric guide components exhibited greater relative material utilization than the grade-4 titanium fixation pins. The maximum predicted guide-component deformation was 0.093 mm. Conclusions: Within the assumptions and limitations of the model, these findings indicate that the mechanical response of the assembly is more strongly influenced by the polymeric guide components, while the distribution of stress between individual guide components depends on the assumed contact scenario.

## 1. Introduction

Oral rehabilitation of patients with terminal or fully failing dentition with implant-supported fixed restorations has advanced considerably over the last decade, driven largely by the digitalization of the diagnostic, planning, custom design and manufacturing workflow [1]. The integration of intraoral scanning, facial scanning, and cone-beam computed tomography (CBCT) into a single dedicated planning environment allows the creation of a virtual dental patient, on which prosthetically driven implant positions can be defined and subsequently transferred to the surgical field through additively manufactured surgical guides [2,3]. Static computer-aided implant surgery is preferred in many settings owing to its predictability, reduced cost, and shorter learning curve relative to dynamic navigation [4].

For the full edentulous arch scenario, the stackable surgical template protocol has emerged as a robust solution that couples immediate, flapless implant placement with the delivery of an immediate fixed provisional restoration [2,5]. The protocol is built around a pin-retained base guide that fits the maxillary dental arch and is fixed with transverse metallic pins; onto this base, a sequence of interchangeable guides is stacked in a defined order: a tooth-supported surgical guide (when residual teeth are still present), an implant osteotomy guide for the remaining osteotomies after extractions, an optional bone-reduction guide, and finally a prosthetic guide for positioning the provisional restoration [2]. The connection between the base and the successive guides is established by matrix–patrix attachments, magnets, balls, screws, or notches, ensuring reproducible repositioning throughout the procedure [2]. Because the entire sequence is referenced to a single fixed and stable base, the accuracy of implant transfer is largely governed by the mechanical stability of that base and of the metallic pins that retain it.

The materials selected for these guides are central to their clinical performance. Polymeric guides are typically produced from biocompatible methacrylate-based photopolymer resins by digital light processing (DLP) or stereolithography (SLA); their flexural behavior, dimensional stability, and fracture resistance directly determine whether the planned implant position is faithfully reproduced [2,6,7]. Chemically, these are not poly(methyl methacrylate) but cross-linked (meth)acrylate networks—commonly based on urethane dimethacrylate (UDMA) or bisphenol-A-derived dimethacrylates (Bis-GMA/Bis-EMA) together with lower-viscosity diluent monomers (e.g., TEGDMA or EGDMA) and a photoinitiator, since the monofunctional methyl methacrylate monomer alone cannot form the three-dimensional network required for vat photopolymerization. Such Class I photopolymers remain the reference materials for 3D-printed surgical guides because they combine adequate rigidity, optical translucency for intraoperative inspection, low cost, and a biocompatibility profile suitable for short-term (<24 h) oral contact—contingent on adequate post-polymerization [8,9,10].

The retention pins, in turn, are manufactured from commercially pure or grade-4 titanium, exploiting the high stiffness, strength, and established osseous biocompatibility of the material. The mechanical connections between the compliant polymeric guides and the stiff metallic pins, transmitted to the residual maxillary bone, define the load path of the whole system during implant bed osteotomy.

Despite the growing clinical adoption of stackable guides, guide fracture during implant-bed preparation remains a recognized intraoperative complication, with reported incidences between 6.7% and 9.7% for guided surgery in general [11]. Finite Element Analysis (FEA) is a well-established numerical method for predicting stress and deformation in complex, patient-specific geometries under defined loading conditions and is increasingly applied in implant dentistry [12,13,14]. In a previous study, we analyzed a single tooth-supported, sleeveless resin guide for the mandible and demonstrated that the material and design could withstand the drilling forces without fracture [11]. However, the biomechanical behavior of the far more complex, multi-component stackable assembly—comprising several polymeric guides and metallic pins loaded simultaneously on an edentulous maxilla—has, to the best of our knowledge, not previously been investigated.

The maxilla presents a specific biomechanical challenge: its predominantly trabecular architecture and thin cortical shell provide less rigid support than the mandible, so a larger share of the drilling load is carried by the guide–pin solution instead of being borne by the bone. The fully edentulous maxilla, lacking dental support entirely, relies solely on mucosal support and pin fixation, making the mechanical adequacy of the guide materials even more decisive [15]. This resilient, purely muco-supported, pin-retained field therefore places the mechanical adequacy of the guide materials at the center of a predictable outcome, since the compliant soft-tissue support must be compensated for by the rigidity of the guide and the anchorage of the transverse pins [16].

The aim of the present study was to evaluate, by means of finite element analysis (FEA), the stress and displacement distribution within the load-bearing components of a stackable surgical guide system during implant osteotomy in a fully edentulous maxilla, with particular emphasis on the mechanical response of the methacrylate-based guides and titanium fixation pins under the selected maximum simulated drilling load. The engineering hypothesis was that the maximum stresses generated in any component of the considered assembly would not exceed the admissible strength of its material, and that the resulting structural deformations would remain limited.

## 2. Materials and Methods

The present investigation was designed as an in silico biomechanical study based on previously acquired clinical imaging and digital data. The anatomical geometry used for the finite element model was derived from the anonymized dataset of a patient included in our previously published case series [2]. In that clinical study, CBCT and intraoral scanning were performed as part of the established diagnostic, treatment-planning, and rehabilitation protocol, and the study was approved by the Ethics Committee of the “Carol Davila” University of Medicine and Pharmacy (Approval No. 36368/2023). Informed consent was obtained from all participants. No patient was prospectively recruited for the present FEA study, and no additional treatment, intervention, imaging examination, or clinical procedure was performed specifically for the current investigation. The objective was to characterize, by means of Finite Element Analysis (FEA), the stress and displacement distribution within a stackable surgical guide assembly under a maximum simulated axial drilling load. The analyzed system comprised four components—the maxillary bone (Maxilla), the polymeric base guide (Base), the implant osteotomy guide (Implants), and the five metallic Fixation pins (Pins). All geometric processing and the analysis itself were performed in CATIA^®^ V5 (Dassault Systèmes, Vélizy-Villacoublay, France), employing the DMU Optimizer, Generative Shape Design, Shape Sculptor, Digitized Shape Editor, Assembly Design, and Part Design workbenches. The methodological workflow followed four important sequential stages: acquisition and reconstruction of the three-dimensional geometry of each component; conversion of the scanned mesh surfaces into solid models; assignment of material properties, assembly, discretization, interference editing, and definition of the physical constraints and loads; and, finally, solution of the analysis and extraction of the results.

### 2.1. Geometry Acquisition and Solid Model Reconstruction

The previously acquired CBCT data (DICOM format) and maxillary intraoral scan (STL format) were imported and superimposed in R2Gate^®^ software, version 2.20 (MegaGen, Daegu, Republic of Korea) as previously described [2]. The geometry of the Maxilla support component used in the present finite element model was derived from the maxillary intraoral scan and therefore reproduced the external edentulous intraoral surface, whereas the CBCT data were used in conjunction with the IOS dataset for prosthetically driven virtual planning and implant positioning. The final restoration was digitally simulated, and six implants were virtually planned. Based on this planning, the stackable surgical guides were designed in Blender for Dental (B4D; Blenderfordental^®^), an add-on running within Blender (Blender Foundation, Amsterdam, The Netherlands), whereas the provisional restoration was designed in Exocad^®^ DentalCAD, version 3.2 Elefsina software (Exocad GmbH, Darmstadt, Germany). A minimum guide wall thickness of 3 mm was maintained during design, consistent with the R2GATE/R2Guide design recommendations and the previously described clinical workflow [2]. Five fixation pins (four transverse and one palatal) were modeled separately in CATIA V5 (Dassault Systèmes, Vélizy-Villacoublay, France) according to the actual simple shapes and dimensions of the pins used clinically, reproducing the number and positions employed during surgery to stabilize and retain the base guide.

From the resulting digital geometry, only the base guide, implant guide, fixation pins, and the main part of the scanned maxilla were included in the finite element analysis; the remaining stackable components and the provisional restoration were excluded from the model. The STL files corresponding to the maxillary model, base guide, and implant guide were verified, corrected, simplified, and converted into three-dimensional solid models in CATIA V5. Due to the high complexity of the three-dimensional scans, each component, including the maxilla, base guide, and implant guide, required careful examination. Each surface was composed of a very large number of triangular elements connected along their adjacent edges and vertices. Although a high element count allows an accurate representation of the three-dimensional geometry and facilitates assembly procedures and the definition of physical constraints between components, it also requires substantial hardware resources.

Therefore, simplification of the three-dimensional models was necessary. CATIA V5 was used to perform a detailed analysis of the element structure, followed by simplification and optimization of each model geometry. For example, in the maxillary model, the number of triangular elements was reduced from 71,994 to 18,464 (Figure 1). Numerous small triangular elements were merged into larger elements while preserving the overall morphology, accuracy, and dimensions of the original three-dimensional model.

Prior to its conversion into a solid model, the maxillary surface was verified for errors and deviations from the initial form, a tangency condition was imposed between adjacent faces, and a further simplification was carried out, transforming the triangular elements into a large number of patches (Figure 2).

These patches accurately covered the entire surface of the maxillary model—in this case, 843 faces were generated—and the surface was additionally checked for overlapping, self-intersecting, or missing regions. A surface detail level of 1000 was imposed (Figure 2), ensuring a high geometric accuracy of the model together with the simplification required for the FEA. Where appropriate, this stage was followed by mesh-smoothing operations, which locally levelled irregularities and peaks (value of Max. surface deviation = 2.024 mm in Figure 2) resulting from the scanning process and from prior editing. The approximately 2.024 mm value represents a localized maximum pointwise deviation identified during an intermediate surface-reconstruction step and should not be interpreted as a uniform deviation of the final maxillary support geometry or as a direct measure of guide–support misfit.

The conversion of this complex surface into a solid 3D model was achieved by closing it along its entire profile. This step is of particular importance, since the solid model is the entity involved in the FEA; any error propagated undetected from the preceding stages may invalidate the whole model (of the respective part and also of the assembly) and compromise the conformity of the results obtained from the analysis.

Once all components had been converted into solid models, material properties were assigned as summarized in Table 1. The maxillary bone was modeled using elastic constants reported for maxillary bone in the biomechanical literature [17,18,19]. The polymeric Base and Implant guides were modeled using the material properties of E-Guide^®^ Resin (EnvisionTEC GmbH, Gladbeck, Germany). E-Guide Resin is a Class I biocompatible, methacrylate-based photopolymer developed for the DLP/SLA additive manufacturing of implant surgical guides [6,11]. Upon polymerization, the material forms a cross-linked (meth)acrylate network intended for short-term (<24 h) intraoral use. The manufacturer-reported elastic properties were therefore assigned to both the Base and Implant guides in the present model (Table 1). The fixation pins were modeled as commercially pure grade 4 titanium (Ti Grade 4; Ti75A). All materials were assumed to be homogeneous, isotropic, and linearly elastic, a commonly adopted simplification in finite element analyses of this type [11,12,13].

As a static linear-elastic analysis was performed, mass density does not influence the computed stress and displacement fields and is reported for completeness [20].

### 2.2. Assembly and Discretization

The components were assembled (Figure 3a–c, with the maxilla shown in transparent representation) in the CATIA V5 Assembly Design workbench, using specific positioning constraints—surface-to-surface contact and coincidence between the axes of the guiding holes and those of the Fixation pins. The correct definition of these constraints is likewise important, as it ensures the consistency of the assembled system.

Each component was discretized into a mesh of tetrahedral finite elements connected through faces, edges, and nodes. The following discretizations were adopted: Maxilla—6075 nodes and 24,090 elements; Base—11,551 nodes and 38,168 elements; Implants—25,162 nodes and 86,527 elements; and Fixation Pins—90 nodes for each pin and 206 elements. The Implants guide received the finest discretization (Figure 4) because it has the highest geometric complexity, it is in contact with all other components except the Pins, and carries the applied loads on some of its surfaces. The Base guide was also finely discretized, since the loads applied to the Implants guide are transmitted through it to the Maxilla. The simplest discretization was applied to the Pins, owing to their simple geometry and reduced dimensions.

Because each component was scanned individually, small interferences between contacting solids were unavoidable. Interference checks were performed between all component pairs, and the small overlapping regions were removed directly within the assembly through Boolean subtraction, which yields correct contact areas between the Maxilla–base, Base–Implant, and Maxilla–Implant pairs (Figure 5).

### 2.3. Physical Constraints and Loads

Physical (contact) constraints were defined on the corresponding contact surfaces between component pairs to transmit and receive the applied loads. These faces were paired manually with particular care so that load transfer and distribution would be reproduced as precisely as possible. The following numbers of physically constrained faces were established: Maxilla 239—Base 339 (eg. in Figure 6); Base 327—Implants 340; Maxilla 72—Implants 127; Base 2—each Pin 2; and Maxilla 1—each Pin 1. The number of paired faces varied according to the geometry and dimensions of the respective components. The superior surface of the Maxilla was fully constrained to prevent displacement of the fixed region during the analysis. This condition is important because that surface is the only fixed area of the proposed assembly.

The modeled support condition represents the base guide after complete seating and stabilization by the five fixation pins. Because the objective was to isolate load transfer through the pin-retained surgical assembly during osteotomy, a distinct mucosal layer was not explicitly modeled. For this specific load case, the pin–support interfaces were treated as the primary direct restraint against gross rigid-body translation and rotation, representing the stabilizing function of fixation pins clinically anchored in bone. The contribution of mucosal compliance to guide seating and local load sharing therefore remained outside the modeled scope. This deliberate abstraction should not be interpreted as implying that mucosal support is clinically negligible.

Two contact scenarios were evaluated: Scenario 1 (idealized no-sliding contact), in which relative sliding was prevented at all paired component interfaces; and Scenario 2 (frictional contact), in which relative sliding was permitted at the Maxilla–Base, Maxilla–implant osteotomy guide, Base–implant osteotomy guide, and Base–fixation pin interfaces, while the Maxilla–fixation pin interfaces remained under a no-sliding condition. For Scenario 2, coefficients of friction of 0.30 were assigned to the Maxilla–Base and Maxilla–implant osteotomy guide interfaces, 0.40 to the Base–implant osteotomy guide interface, and 0.35 to the Base–fixation pin interfaces.

The coefficient of friction at each interface may be influenced by material pairing, surface finish, build orientation, dimensional tolerances, lubrication, and biological conditions. Intraoperatively, blood, saliva, and sterile irrigation fluid may reduce friction relative to dry contact. Because experimentally determined coefficients were unavailable for the specific resin–resin and titanium–bone interfaces involved in the clinical workflow, as well as for the clinical resin–mucosa interface, the values used in the model were adopted from indirect tribological data for comparable material classes and treated as literature-informed engineering assumptions rather than interface-specific constants. Metallic surfaces sliding against bone have been reported to exhibit coefficients of approximately 0.3–0.5, with titanium values commonly around 0.4 [21,22], whereas 3D-printed polymer contacts exhibit coefficients within a comparable range that generally decrease under wet or lubricated conditions [23,24]. Accordingly, the coefficients assigned to the modeled Maxilla–guide interfaces should not be interpreted as reproducing actual resin–mucosa friction. The frictional formulation was therefore used as a comparative modeling scenario to examine how permitting relative interface motion influences load transfer within the assembly, without implying that it reproduces the exact intraoral contact environment.

The loaded guide hole corresponded to a peripheral/terminal osteotomy site and was selected because its eccentric position relative to the pin-retained base was expected to generate a comparatively unfavorable rotational tendency under the applied load. Two equivalent loads were applied to the implant osteotomy guide. The first consisted of a distributed axial force of 10.14 N (derived in Section 2.4), applied to the chamfered and cylindrical surfaces of the guide hole corresponding to the region of drill complete engagement (Figure 7a). The force was directed coincident to the axis of the guiding hole. The second consisted of a torsional moment of 3.5 × 10^−5^ N·m, applied about the same axis (Figure 7b). Both values were derived from the drilling-regime relations and experimentally determined coefficients previously used for a Ø2 mm drill in maxillary bone [11], accounting for feed, cutting speed, bone hardness, and drilling conditions. These values represent the calculated axial cutting force and cutting torque generated at the drill–bone interface. For the present study, they were deliberately transferred to the guide hole as conservative equivalent loads representing full transmission of the drilling demand to the guiding component during drill engagement. This constitutes an upper-bound modeling assumption rather than a direct measurement of drill–guide contact forces. Actual load transfer to the guide depends on factors such as drill-to-guide clearance, eccentricity, runout, local contact conditions, and friction, which were not independently quantified in the present study. The forces associated with seating and assembling the guide components on the maxilla, the forces generated during insertion of the fixation pins, and any assembly induced preloads or residual contact forces that may develop after positioning and fixation of the guide or between its individual components were not included in the analysis. These loads could not be reliably determined or quantified within the present modeling framework and were therefore not incorporated into the finite element model.

The complete methodology for calculating the recommended drilling parameters is presented below, with values computed for two scenarios: spindle speeds of 750 and 1200 rev/min.

### 2.4. Calculation of Osteotomy Drilling Process Parameters

Feed, mm/rev, it is the axial advance of the drill into the Maxilla per revolution

*f* = *K_f_* × *C_f_* × *d*^0.6^ mm/rev,

where: *K_f_* is a correction coefficient dependent on the hole depth and its diameter, for l > 3d; 10 mm > 3 × 2 mm; *K_f_* = 0.9; *C_f_* is a feed coefficient that accounts for the HB hardness of the maxillary bone and the drilling accuracy, *C_f_* = 0.065; *d* is the drill diameter, *d* = Ø2 mm

f = 0.9 × 0.065 × 2^0.6^ = 0.09 mm/rev

Feed rate, mm/min, it is the speed at which the drill advances linearly into the Maxilla

*V_f_* = *f* × *n* mm/min,

where: *f* is the feed per revolution of the drill, *n* is the required rotational speed for drilling (between 750 rev/min and 1200 rev/min rotational speed)

*V*_*f*750_ = 0.09 × 750 = 67.5 mm/min

*V*_*f*1200_ = 0.09 × 1200 = 108 mm/min

Cutting speed, m/min, it is the relative linear speed between the outer edge of the rotating drill and the Maxilla. It is typically measured in meters per minute (m/min). Optimal cutting speed prevents the drill bit from overheating, which extends tool life, ensures high-quality hole finishes and reduces the trauma received by the Maxilla.

*V_c_* = π × *d* × *n*/1000 m/min,

where: *d* is the drill diameter; *n* is the required rotational speed for drilling (between 750 rev/min and 1200 rev/min)

*V*_*c*750_ = π × 2 × 750/1000 = 4.71 m/min

*V*_*c*1200_ = π × 2 × 1200/1000 = 7.54 m/min

Axial cutting force, N, (often referred to as the thrust force), it is the force acting longitudinally along the axis of the drill, pushing the drill bit into the Maxilla and compresses the drill along its axis.

*F_fw_* = *C*_*F*1_ × *d^xF^* × *f^yF^* × *HB^nF^* × *K_F_* N,

where: *C*_*F*1_ is an experimentally determined coefficient dependent on the hardness of the maxillary bone, *C*_*F*1_ = 1.15; *d* is the drill diameter, *d* = Ø2 mm; *xF* and *yF* are experimentally determined correction coefficients dependent on the hardness of the maxillary bone and the drilling conditions (guided; with/without cooling), *xF* =1, *yF* =0.65; *f* is the feed per revolution of the drill, *f* = 0.09 mm/rev; HB is the mean hardness of the maxillary bone, HB = 45 HB; *nF* is an experimentally determined coefficient dependent on the drill type and the hardness of the maxillary bone, *nF* = 0.75; *K_F_* is a correction coefficient for the drilling force, calculated as:

*K_F_* = *K_aF_* × *K_faF_* × *K_χF_* × *K_ηF_*,

where: *K_aF_* is a correction coefficient dependent on the drill grinding (point) geometry, *K_aF_* = 1; *K_faF_* is an experimentally determined correction coefficient dependent on the manner in which the drill is fed manually, *K_faF_* = 1; *K_χF_* is a correction coefficient dependent on the value of the drill point angle 2χ^0^, *K_χF_* = 0.98; *K_ηF_* is a correction coefficient dependent on the relative core thickness of the drill *η* and its diameter, *d*= Ø2 mm, *η* = 0.19, *K_ηF_* = 1.25

*K_F_* = 1 × 1 × 0.98 × 1.25 = 1.23

*F_fw_ =* 1.15 × 2^1^ × 0.09^0.65^ × 45^0.75^ × 1.23 = 10.14 N

Cutting torque, N·mm, it is the rotational force required by a drill bit to cut into and shear through Maxilla. It is a critical parameter that dictates the machine power required and directly impacts drill bit wear, hole quality, and process stability.

*M_W_* = *C*_*M*1_ × *d^xM^* × *f^yM^* × *HB^nM^* × *K_ηM_* N·mm,

where: *C*_*M*1_ is an experimentally determined coefficient dependent on the hardness of the maxillary bone, *C*_*M*1_ = 0.02; *d* is the drill diameter, *d* = Ø2 mm; *xM* and *yM* are experimentally determined correction coefficients dependent on the hardness of the maxillary bone and the drilling conditions (guided; with/without cooling), *xM* =1.1, *yM* =0.9; HB is the mean hardness of the maxillary bone, HB = 45 HB; *nM* is an experimentally determined coefficient dependent on the drill type and the hardness of the maxillary bone, *nM* = 0.48; *K_ηM_* is a correction coefficient for the drilling torque, *K_ηM_* = 1.15

M_W_ = 0.02 × 2^1.1^ × 0.09^0.9^ × 45^0.48^× 1.15 = 3.5 × 10^−5^ N·m

The calculated value represents the cutting torque generated during drill–bone interaction. In the finite element model, this torque was applied to the guide hole as an equivalent torsional load to represent a conservative condition of full load transfer to the guiding component. It should therefore not be interpreted as a directly measured frictional torque between the drill and the guide.

Before launching the analysis, the entire system was verified (Figure 8): the geometric correctness of every component, the presence of a discretization mesh on each component, the assignment of materials, and the physical connections/constraints between components were all checked.

The finite element analysis was subsequently performed under all previously defined boundary and loading conditions. During the solution process, CATIA V5 conducts complex computations of load distribution across the assembled components, enabling the extraction of the resulting stress, displacement, and deformation fields.

### 2.5. Statistical Analysis

This study used deterministic finite element simulations. No repeated biological or experimental observations were generated; therefore, inferential statistical testing was not applicable. Numerical outputs are reported descriptively as maximum stress, displacement, and utilization values for the predefined contact scenarios.

## 3. Results

Figure 9 presents an overall view of the analyzed system after the solution process, illustrating the correct positioning of the components and the finite element discretization, with the Implants component exhibiting the most complex mesh.

The von Mises stress distribution on each component is presented in Figure 10a–d. Regions of high stress appear in green, yellow, and red in localized areas, arising from the contacts between components and from the loads applied to the Implants component.

Each representation in Figure 10 also displays the load-application region. In the case of the Maxilla, maximum stresses are observed in a localized region corresponding to the applied drilling load. For the Base component, high stresses occurred at the load-transfer region and near one Fixation pin interface, consistent with transmission of the applied torsional load. This Fixation Pin exhibited the highest stress among the Fixation Pins, as shown in Figure 10d, reflecting its role in stabilizing the Base against rotation. The Implants component exhibited its highest stresses in the load-application region and at its interface with the Base component.

Under Scenario 1 (Figure 10a–d), the maximum von Mises stresses for each component were: Maxilla, 2.34 MPa; Base, 3.18 MPa; Implants, 4.32 MPa; and Fixation Pins, between 1.13 and 3.26 MPa. All of these values are much lower than the admissible strengths of the materials assigned to the respective components (Table 1). A summary of the results in relation to the admissible strength of each material is given in Table 2.

The maximum predicted deformations were 0.00086, 0.026, 0.074, and 0.010 mm for the maxillary support, base guide, implant osteotomy guide, and fixation pins, respectively, under Scenario 1, and 0.0011, 0.093, 0.051, and 0.012 mm under Scenario 2.

Figure 11 presents the deformation vectors of the components under the conditions considered in Scenario 1. The highest predicted deformations occurred in the implant osteotomy guide (0.074 mm) and the base guide (0.026 mm), whereas the maxilla and grade-4 titanium fixation pins exhibited substantially smaller deformations because of their higher stiffness. These values represent the structural deformation predicted by the present FEA under the selected loading conditions.

The results presented above correspond to Scenario 1. The results obtained under Scenario 2 are presented below and compared with those of Scenario 1.

Figure 12 illustrates representative examples of the frictional contact configuration implemented for Scenario 2, as defined in Section 2.3.

Under the selected frictional contact formulation, the calculated stresses in all modeled components remained below their respective reference strength values.

Under Scenario 2, permitting relative sliding at the selected interfaces altered the stress and displacement distributions compared with Scenario 1.

Following the new analysis in scenario 2, the maximum von Mises stresses for each component were: Maxilla, 2.48 MPa; Base, 7.63 MPa; Implants, 2.38 MPa; and Fixation Pins, between 1.11 and 3.94 MPa. All of these values are presented in Table 2 and are also much lower than the admissible strengths of the materials assigned in Table 1.

It is observed that the Maxilla exhibits stress levels similar to those in the previous analysis, with an increase in stress within the Base component but a decrease in the Implants. Essentially, the Base component—being precisely assembled onto the Maxilla—absorbs the majority of the stress resulting from the new friction conditions established between the components of the system under study.

Figure 13 presents the stress and displacements vector distributions for the Base component. An increased extent of the regions exhibiting maximum stress can be observed, while their location remains essentially unchanged, as shown in Figure 10b.

Comparison of Scenario 1 (idealized no-sliding contact) and Scenario 2 (frictional contact) demonstrated that the assumed interface behavior influenced the distribution of stress and displacement within the assembly. Despite these differences, both scenarios showed a consistent overall mechanical response and provided complementary information regarding the behavior of the structural design, assembly configuration, and selected materials under the simulated loading conditions.

Across both scenarios, the calculated von Mises stresses remained below the selected reference strength values under the modeled loading and boundary conditions, with the polymeric guide components exhibiting the greatest relative material utilization. Under the idealized no-sliding condition, the base guide and implant osteotomy guide reached 5.3% and 7.2% of their respective reference strength values, whereas under the frictional-contact formulation the base guide exhibited the highest polymer utilization (12.7%). In contrast, the grade-4 titanium fixation pins remained comparatively lightly stressed in both contact formulations. Across both contact scenarios, the maximum predicted deformation of the polymeric guide components was 0.093 mm, occurring in the base guide under Scenario 2, while the implant osteotomy guide reached a maximum deformation of 0.074 mm under Scenario 1. These values represent the structural deformation predicted by the present FEA under the selected loading and boundary conditions and should not be interpreted as a direct measure of clinical implant-placement accuracy.

Clinical cracking or fracture may involve mechanisms not represented in the present model, including material defects, assembly misfit, cyclic loading, and other unmodeled loading conditions; these mechanisms cannot be evaluated by the present linear-elastic analysis.

## 4. Discussion

To the best of our knowledge, the present study is the first to evaluate the biomechanical behavior of the surgical-phase load-bearing components of a multi-component stackable surgical guide assembly designed for flapless, prosthetically driven rehabilitation of a fully edentulous maxilla. The study hypothesis was supported within the assumptions of the present model and under the selected simulated drilling conditions: with an axial force of 10.14 N and an equivalent torsional load derived from the calculated cutting torque, the maximum von Mises stresses in all modeled components remained below their respective reference strength values in both contact scenarios. The distribution and magnitude of stress and displacement, however, varied according to the assumed interface behavior, with the reported deformations of the polymeric guide components remaining ≤0.093 mm across the two scenarios.

The most biomechanically relevant finding concerns the role of the guide materials within the load path. Although the grade-4 titanium fixation pins were by far the stiffest and strongest components (Young’s modulus 115 GPa; reference strength 550 MPa), they exhibited very low relative material utilization in both scenarios, with maximum stresses of 3.26 MPa in Scenario 1 and 3.94 MPa in Scenario 2, corresponding to less than 1% of their reference strength. In contrast, the polymeric guide components exhibited substantially greater relative material utilization. Under Scenario 1, the implant osteotomy guide reached a maximum von Mises stress of 4.32 MPa, corresponding to 7.2% of the 60 MPa reference strength of the methacrylate-based photopolymer, whereas under Scenario 2 the highest polymer stress occurred in the Base guide, reaching 7.63 MPa, or 12.7% of the reference strength. Thus, the location of the highest polymer stress depended on the assumed contact formulation, emphasizing the influence of interface behavior on load transfer within the stackable assembly.

These findings indicate that, within the assumptions of the present linear-elastic model, the mechanical response of the assembly is more strongly influenced by the polymeric guide components than by the titanium fixation pins. The more compliant polymeric guides accommodate a greater proportion of the deformation associated with the applied drilling load, whereas the rigid fixation pins primarily contribute to stabilization of the assembly. From a materials perspective, this identifies the mechanical properties of the printable resin as an important consideration for future optimization of the guide system. Printable resins with enhanced flexural strength and fracture toughness, including reinforced formulations, may therefore warrant further investigation for this application. However, because alternative resin formulations were not evaluated in the present model, the potential mechanical benefit of such modifications should be regarded as a hypothesis requiring dedicated numerical and experimental validation.

These results are consistent with, and extend, our previous FEA of a single tooth-supported, sleeveless resin guide for the mandible, in which a maximum stress of 6.92 MPa and a maximum displacement of 0.002 mm were obtained for an isolated guide loaded by a Ø2 mm drill [11]. In the present multi-component system, the mechanical response was scenario-dependent: the highest polymer stress was 4.32 MPa in the implant osteotomy guide under Scenario 1 and 7.63 MPa in the base guide under Scenario 2, while the maximum reported guide-component deformation reached 0.093 mm. Rather than demonstrating a uniformly lower peak stress, these findings indicate a redistribution of stresses and greater deformation compared with the previously investigated tooth-supported configuration. This difference likely reflects the fundamentally different mechanical conditions of the two models: a multi-component assembly on an edentulous maxilla lacks the rigid tooth support available in the partially dentate mandible, and the applied load is transferred through several interconnected guide components and fixation interfaces.

The maxilla was, moreover, assigned a lower homogenized elastic modulus than that used for dense mandibular cortical bone in the previous model, providing a less rigid supporting structure within the present numerical formulation. These differences in geometry, support conditions, material assignment, and assembly configuration limit direct quantitative comparison between the two studies but may contribute to the greater displacements observed in the present model. The maximum von Mises stress predicted in the maxillary support component was 2.34 MPa in Scenario 1 and 2.48 MPa in Scenario 2. Because bone damage or failure criteria were not incorporated into the present linear-elastic model, these values should be interpreted as comparative stress outputs rather than as evidence of a specific biological safety threshold.

The findings also provide considerations for future design optimization of the stackable protocol itself. The component exhibiting the highest polymer stress differed between the two contact scenarios: the implant osteotomy guide was most highly utilized in Scenario 1, whereas the base guide showed the highest utilization in Scenario 2. This finding suggests that future parametric analyses should evaluate both local guide geometry and interface behavior, including the influence of wall thickness around the guiding cylinders, connection geometry, and material distribution on stress concentrations.

From a practical design and manufacturing perspective, several additional factors may warrant consideration in future investigations. A small mechanical clearance between mating printed components (approximately 0.05–0.1 mm) may help accommodate manufacturing tolerances and facilitate seating, while irrigation at the component interfaces may modify frictional behavior during assembly. Build orientation may also influence surface quality and friction at mating interfaces, with vertically printed surfaces reported to exhibit smoother sidewalls and lower friction than surfaces printed at other orientations [25,26]. These factors were not parametrically evaluated in the present study and should therefore be regarded as considerations for future experimental and numerical investigation and not as recommendations directly supported by the current FEA.

The very low relative stress utilization of the titanium fixation pins in both scenarios indicates a substantial mechanical margin under the specific modeled conditions. However, pin diameter, number, position, and distribution were not parametrically varied in the present analysis; consequently, the present findings cannot establish an optimal pin configuration or demonstrate that pin dimensions or number can be reduced without affecting assembly stability.

The clinical rationale for limiting the number of transverse pins—more pins do not necessarily improve stabilization and, in dense bone, may introduce internal stresses [2]—provides useful clinical context for interpreting the present numerical findings. This is consistent with the recent systematic review of static fully guided surgery in fully edentulous patients by Azevedo et al. [16], in which all included clinical studies stabilized the guide with fixation pins or screws—most commonly three or four—yet the authors concluded that guide stability depends more on an even distribution of the pins throughout the arch and on their correct seating than on their absolute number [16]. Notably, that review also reported clinical evidence that fixation pins do not necessarily enhance template stability, and that, when the guide is not correctly seated from the outset, fixation may even perpetuate or amplify the initial malposition [16]. These clinical observations provide useful context for interpreting the present FEA results. In both contact scenarios, the fixation pins exhibited less than 1% utilization of their reference strength, suggesting that their material strength provided a substantial reserve under the simulated loading conditions. However, because pin number, diameter, position, and spatial distribution were not varied in the present model, the current findings should not be interpreted as defining an optimal fixation strategy. Further parametric and experimental studies are required to determine how these variables influence guide stability and load transfer within the assembly.

From a materials perspective, the choice of a methacrylate-based photopolymer for the guides represents a balance among rigidity, translucency for intraoperative inspection, dimensional accuracy, biocompatibility, and cost [2,6,11]. The present analysis characterizes the relative mechanical utilization of this material within a multi-component assembly under the selected simulated drilling conditions, with maximum utilization ranging from 7.2% in the implant osteotomy guide under Scenario 1 to 12.7% in the base guide under Scenario 2. This provides a rationale for further investigation of reinforced or biomimetically designed printable resins with higher flexural strength and toughness, and to characterize their behavior not in isolation but within the loaded, multi-component assembly in which they are actually used. The FEA framework presented here may provide a useful platform for comparative in silico screening of candidate materials, provided that material-specific properties are incorporated and the numerical predictions are subsequently validated experimentally.

Several limitations should be acknowledged. First, the present in silico FEA used homogeneous, isotropic, and linearly elastic material models. The additively manufactured resin was represented by bulk material properties, whereas printed components may exhibit anisotropy, build-direction dependence, post-processing effects, and manufacturing defects; consequently, comparison of the calculated von Mises stresses with nominal reference strength values should be interpreted as an engineering screening comparison rather than as a validated failure criterion. Accordingly, the present analysis does not predict crack initiation, plastic deformation, fatigue failure, or clinical fracture.

Second, a single patient-derived edentulous maxillary geometry and one selected static drilling condition for a Ø2 mm osteotomy were analyzed. Dynamic and cyclic loading, larger-diameter drills, off-axis forces, implant insertion, repeated instrumentation, and fatigue were not modeled. The findings therefore characterize the response of the investigated configuration under a specific loading condition and should not be generalized to the complete range of forces that may occur during clinical implant surgery.

Third, the support conditions were intentionally simplified. Clinically, the base guide is seated on resilient mucosa and subsequently stabilized by fixation pins. The present model isolates the pin-retained load path after complete fixation; for this specific modeled condition, the pin–bone interfaces were treated as the primary direct restraint against gross rigid-body motion under the applied drilling reaction load. This does not imply that mucosal support is clinically negligible, since mucosal thickness and compliance may influence seating, contact pressure, local load sharing, and absolute displacement. Because the mucosal layer was not explicitly represented, the modeled Maxilla–guide interfaces do not reproduce the actual resin–mucosa contact mechanics and may overestimate assembly stiffness. Accordingly, the modeled maxillary support should be regarded as an idealized structural representation rather than as a complete simulation of the clinical tissue–guide interface.

Fourth, the friction coefficients used in Scenario 2 were literature-informed engineering assumptions rather than experimentally measured interface-specific values. No parametric friction-coefficient sensitivity analysis was performed; therefore, the extent to which alternative interface coefficients would modify stress and displacement cannot be determined from the present results. In addition, Boolean-edited conforming surfaces represented a nominally manufactured and correctly seated assembly and did not reproduce dimensional misfit, incomplete seating, or manufacturing variability. The influence of fit tolerance and interface misfit on load transfer therefore remains to be quantified.

Finally, the calculated drill–bone cutting force and torque were transferred to the guide hole as conservative equivalent loads representing full load transmission. These loads should therefore be regarded as an engineering approximation of the loading conditions and should not be interpreted as experimentally measured drill–guide interaction forces.

Future investigations should incorporate patient-specific soft-tissue properties, interface-specific tribological characterization, parametric analyses of guide geometry and fixation configuration, quantitative mesh-convergence and fit-sensitivity analyses, broader loading scenarios, additive-manufacturing anisotropy, and experimental mechanical validation.

## 5. Conclusions

Within the limitations of this in silico study, the following conclusions can be drawn:

(1) Under the selected simulated axial drilling force of 10.14 N and the associated equivalent torsional load derived from the calculated cutting torque, the maximum von Mises stresses in all modeled components remained below their selected reference strength values under both contact scenarios.
(2) The distribution of stress within the polymeric guide components depended on the assumed contact formulation. The implant osteotomy guide exhibited the highest polymer utilization under Scenario 1 (idealized no-sliding contact; 4.32 MPa, 7.2% of the reference strength), whereas the base guide exhibited the highest utilization under Scenario 2 (frictional contact; 7.63 MPa, 12.7%). Across both scenarios, the polymeric guide components exhibited substantially greater relative material utilization than the grade-4 titanium fixation pins.
(3) The maximum predicted guide-component deformation across both contact scenarios was 0.093 mm, occurring in the base guide under Scenario 2. These values represent structural deformation predicted by the present FEA and should not be interpreted as a direct measure of clinical implant-placement accuracy.
(4) The findings indicate that the mechanical response of the assembly was more strongly influenced by the polymeric guide components, while the distribution of stress and displacement was sensitive to the assumed interface behavior.

These findings should be interpreted as an engineering assessment under the specified modeling and loading assumptions. Experimental mechanical validation and further sensitivity analyses are required before specific clinical or design recommendations can be established.

## Figures and Tables

**Figure 1:**
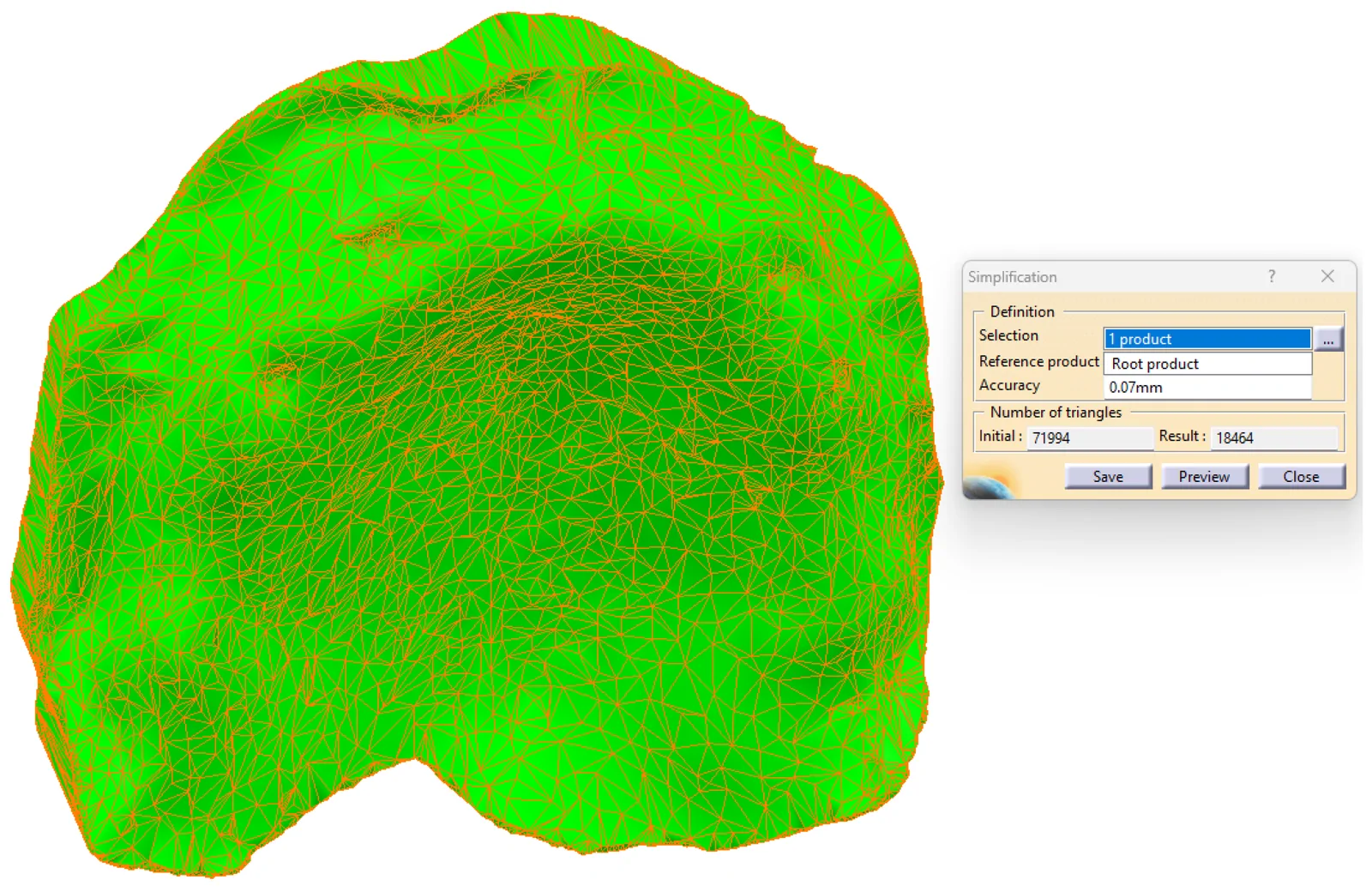
Simplification and optimization of the 3D surface model of the maxilla. The green color overlaying the 3D maxillary model indicates good agreement and consistency with the reference model.

**Figure 2:**
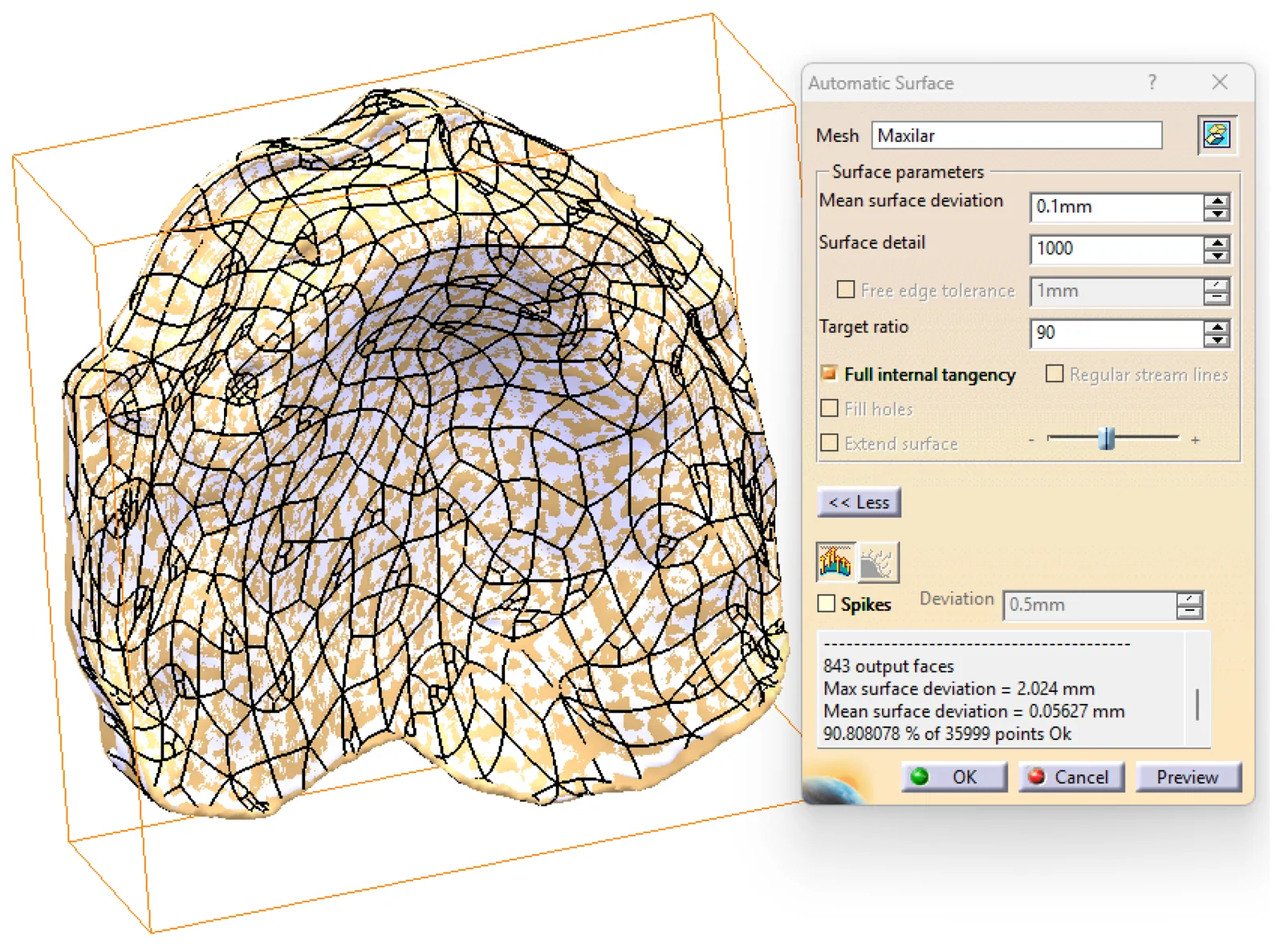
Verification and generation of the final surface of the maxilla.

**Figure 3:**
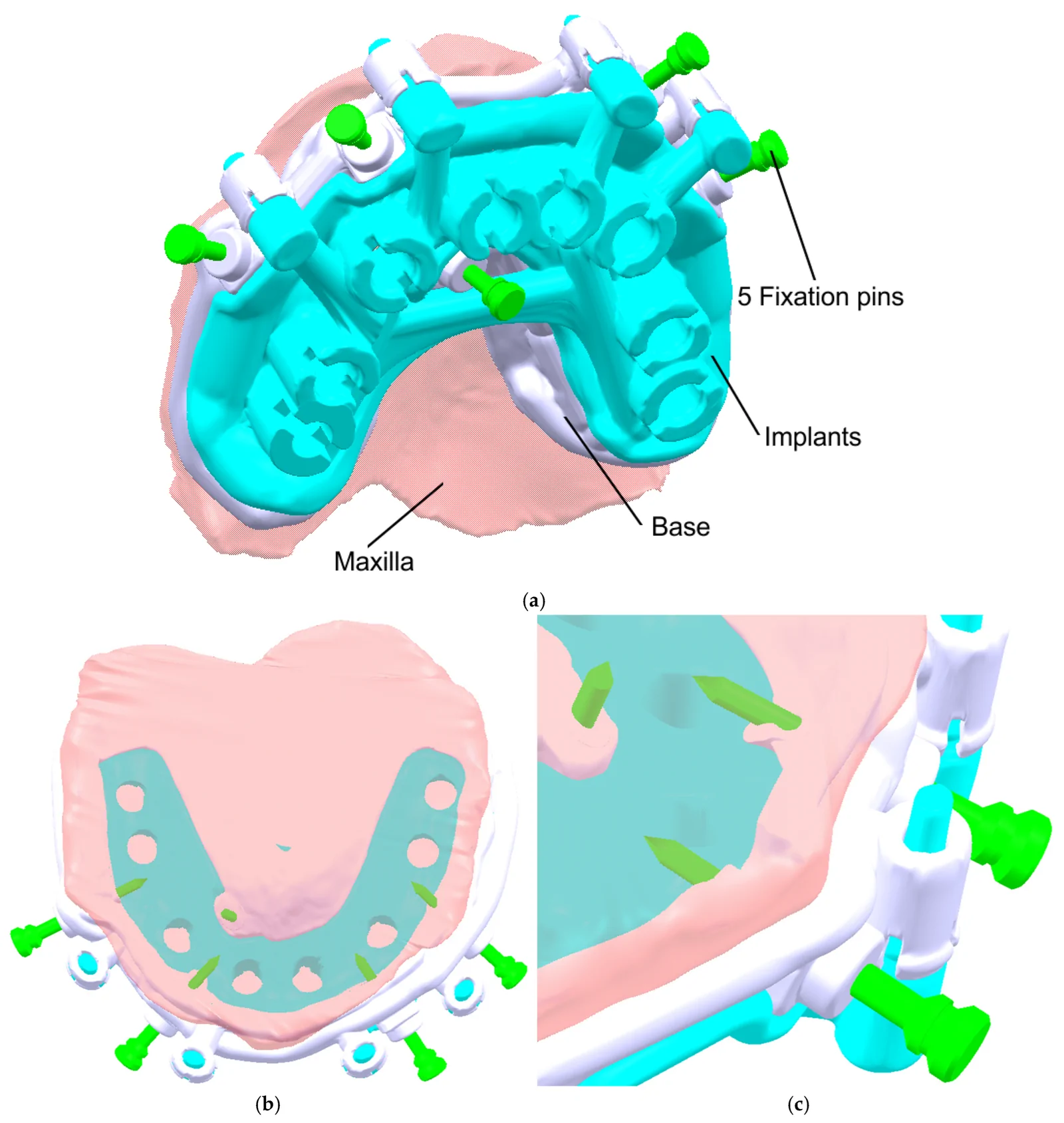
Assembled representation of the analyzed system (Maxilla shown transparent): (**a**,**b**) overall view of the assembled components; (**c**) detail of the assembly of the Fixation pins with the implant guide and the Maxilla.

**Figure 4:**
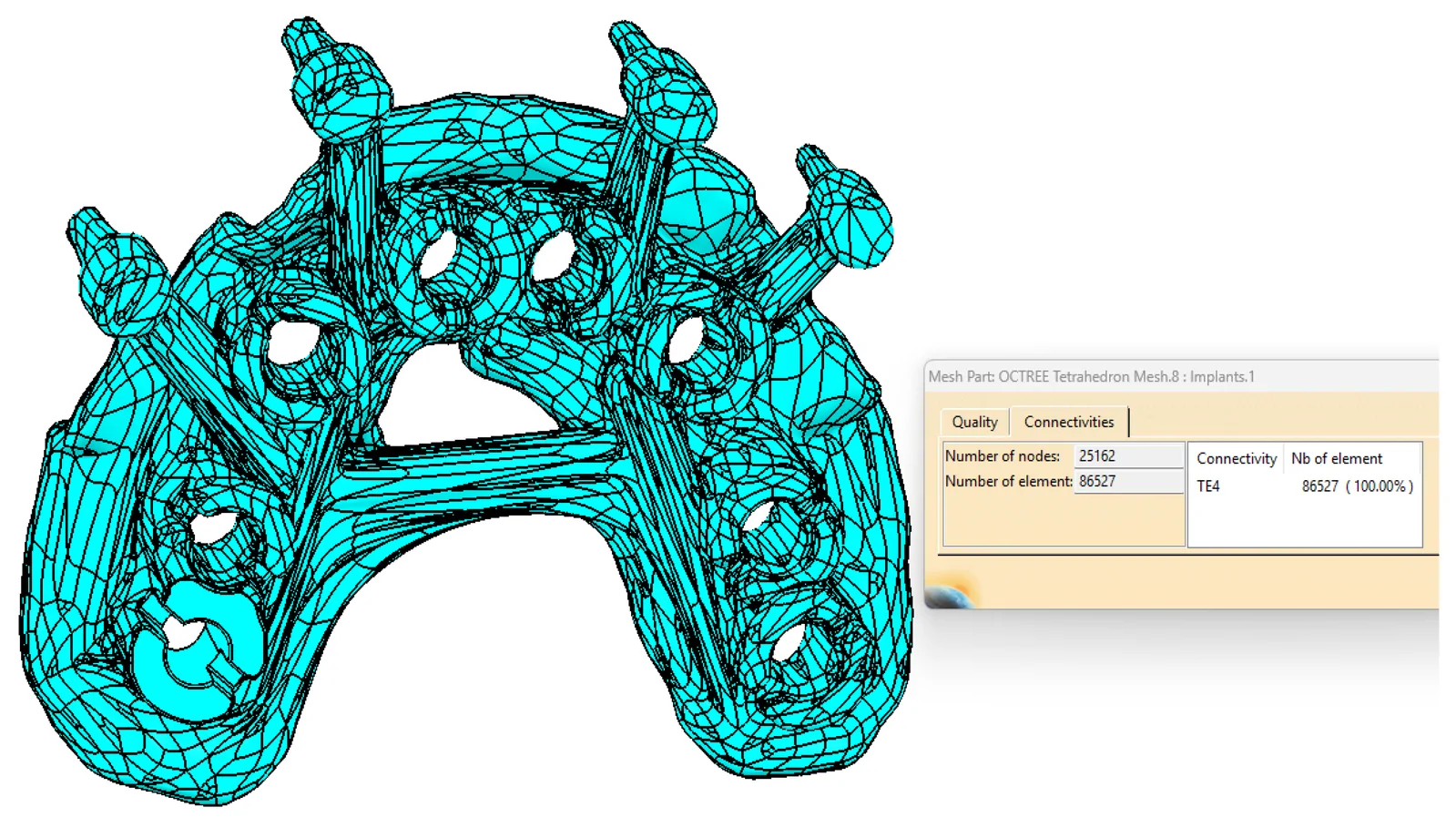
Discretization of the Implants component, which received the finest mesh owing to its geometric complexity and its role in transmitting the applied loads.

**Figure 5:**
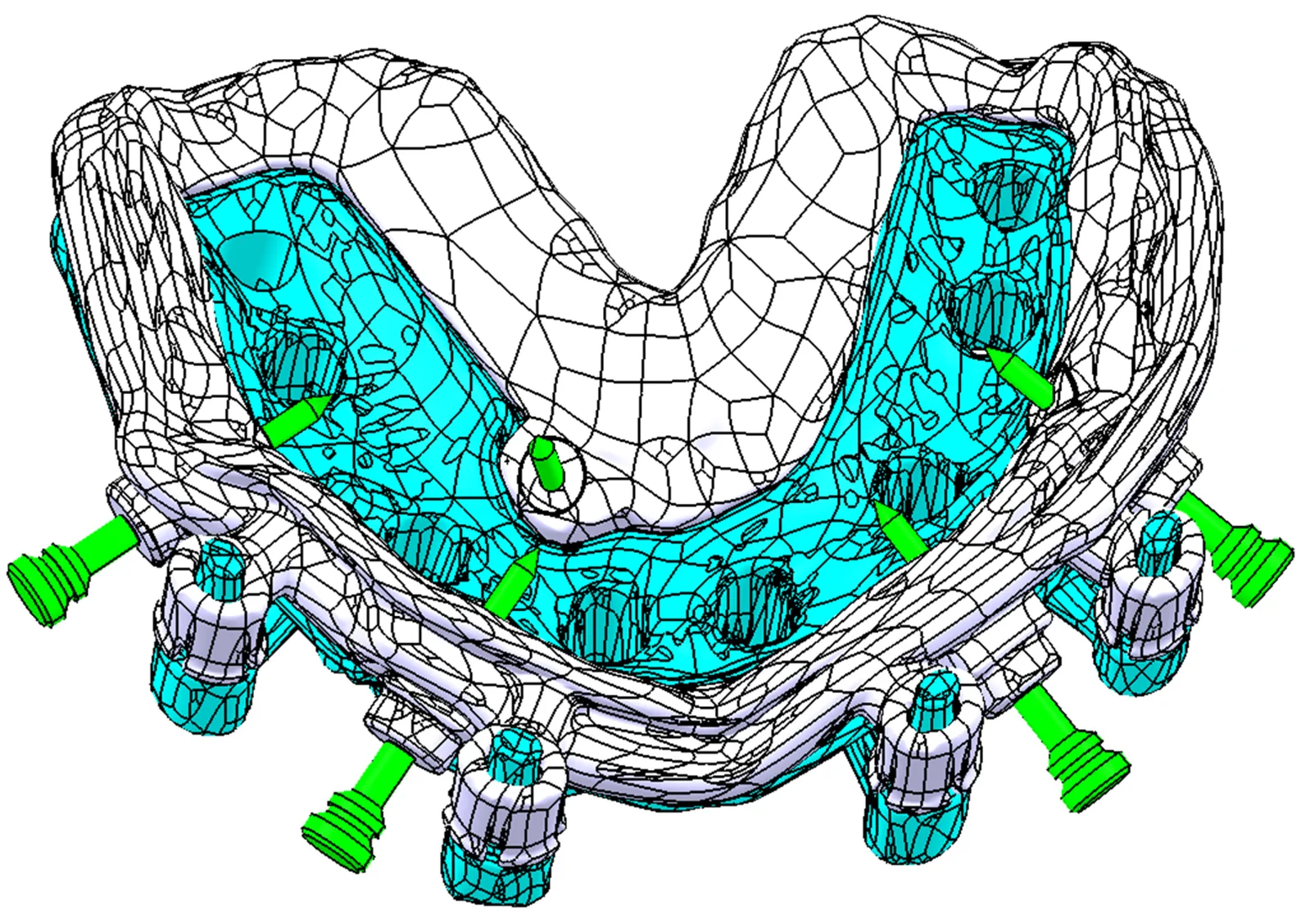
Components attached to the maxilla after interference editing; the contact surfaces between the maxilla, base, and implants guides are visible.

**Figure 6:**
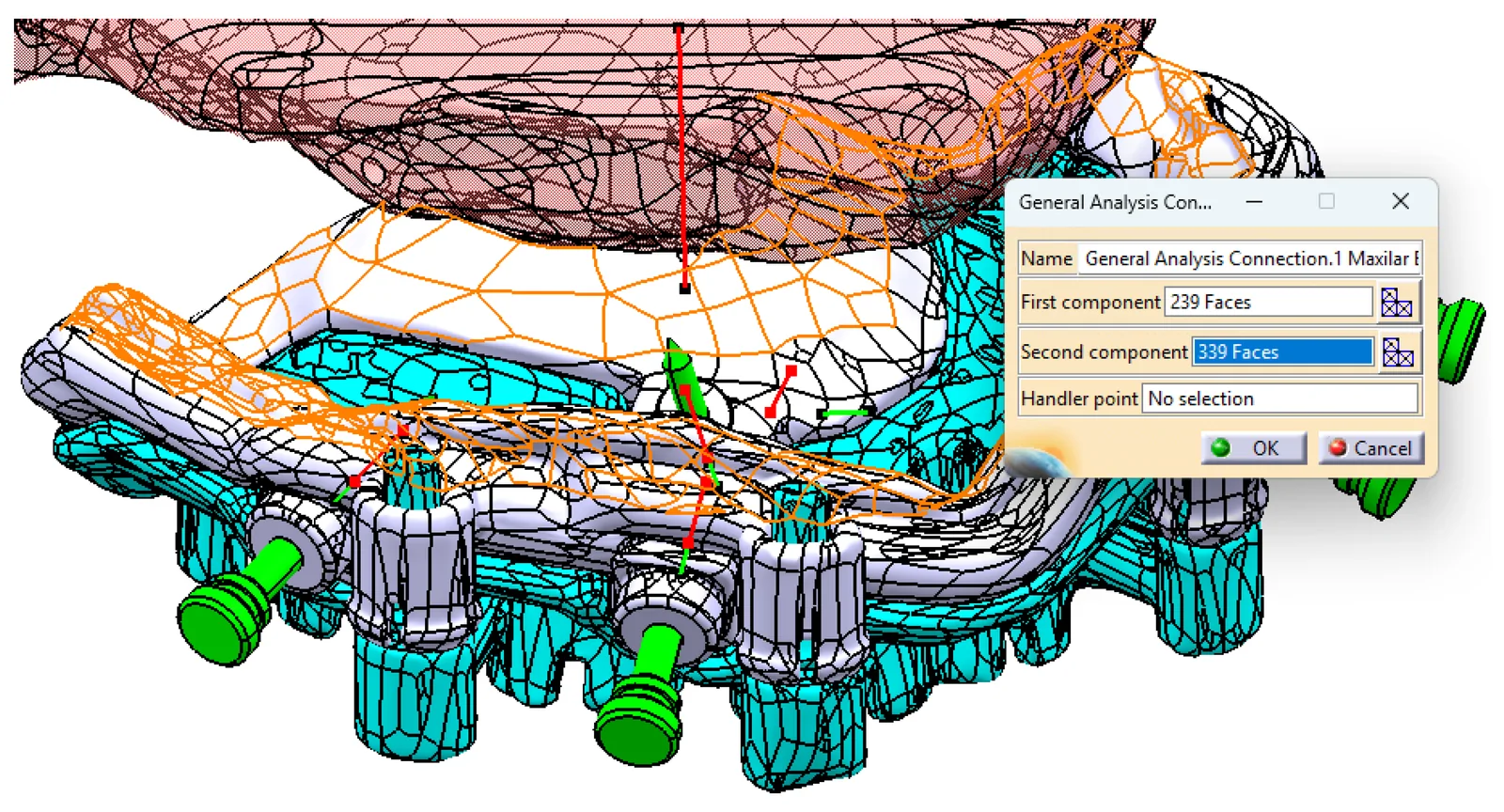
Selection of the contact surfaces between the Maxilla (239 faces) and Base (339 faces) components.

**Figure 7:**
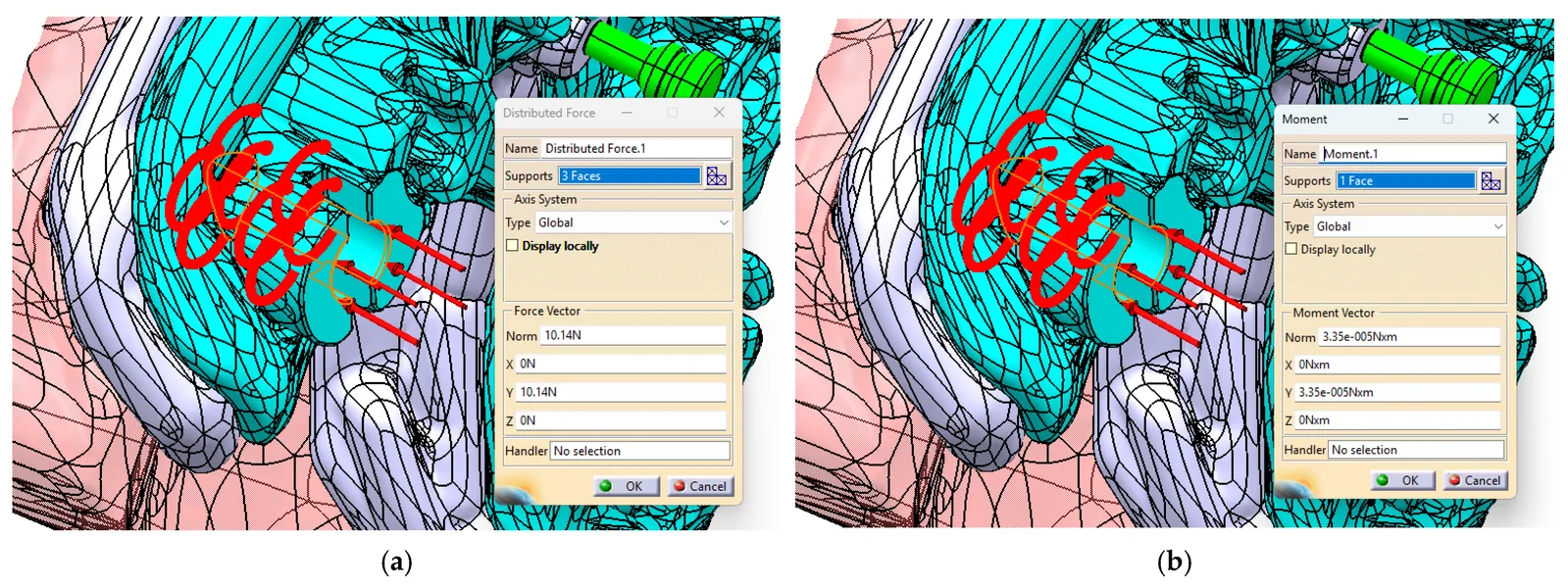
Equivalent drilling loads applied to the implant osteotomy guide: (**a**) distributed axial force of 10.14 N applied to the chamfered and cylindrical surfaces of the guide specified hole, parallel to the hole axis; (**b**) equivalent torsional moment of 3.5 × 10^−5^ N·m applied about the guide-hole axis. The loads correspond to the calculated axial cutting force and cutting torque generated at the drill–bone interface and were transferred to the guide hole as conservative equivalent loads.

**Figure 8:**
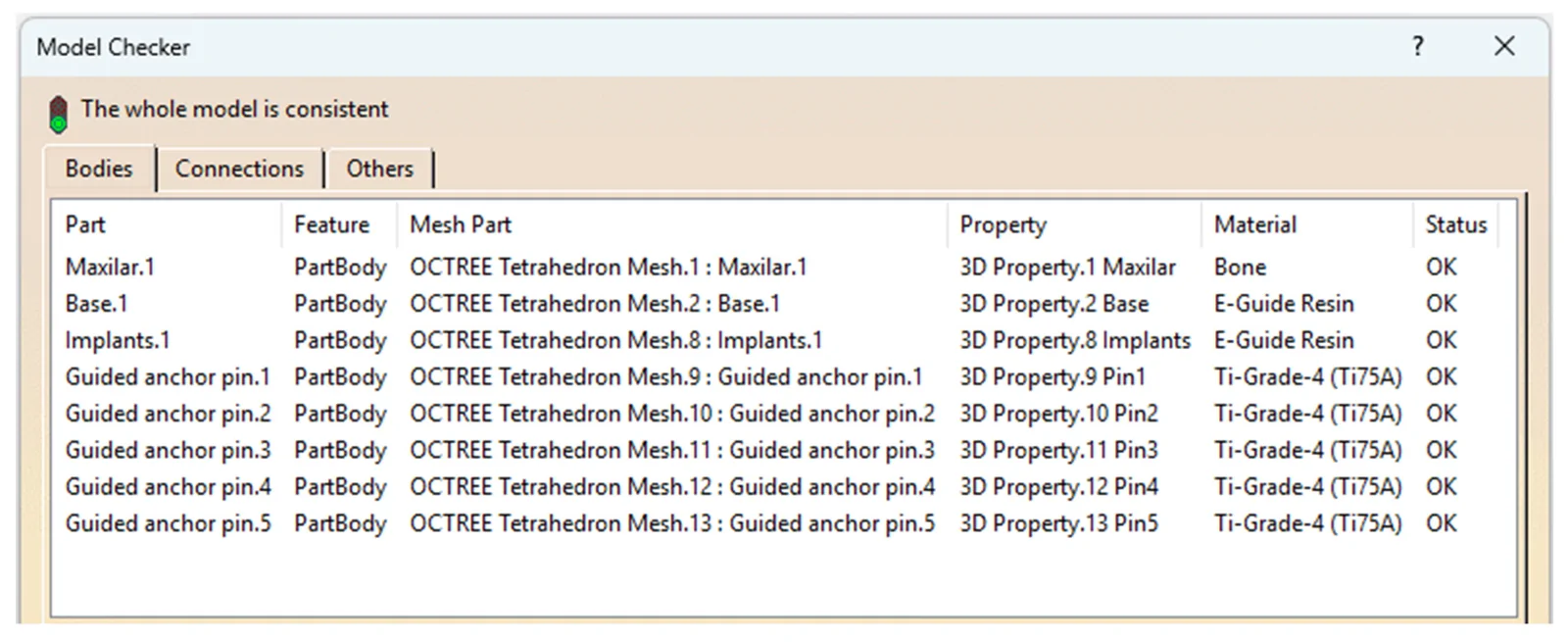
Verification of the whole assembly (maxilla, guide and pins) prior to launching the finite element analysis (component geometry, discretization, materials, and physical constraints).

**Figure 9:**
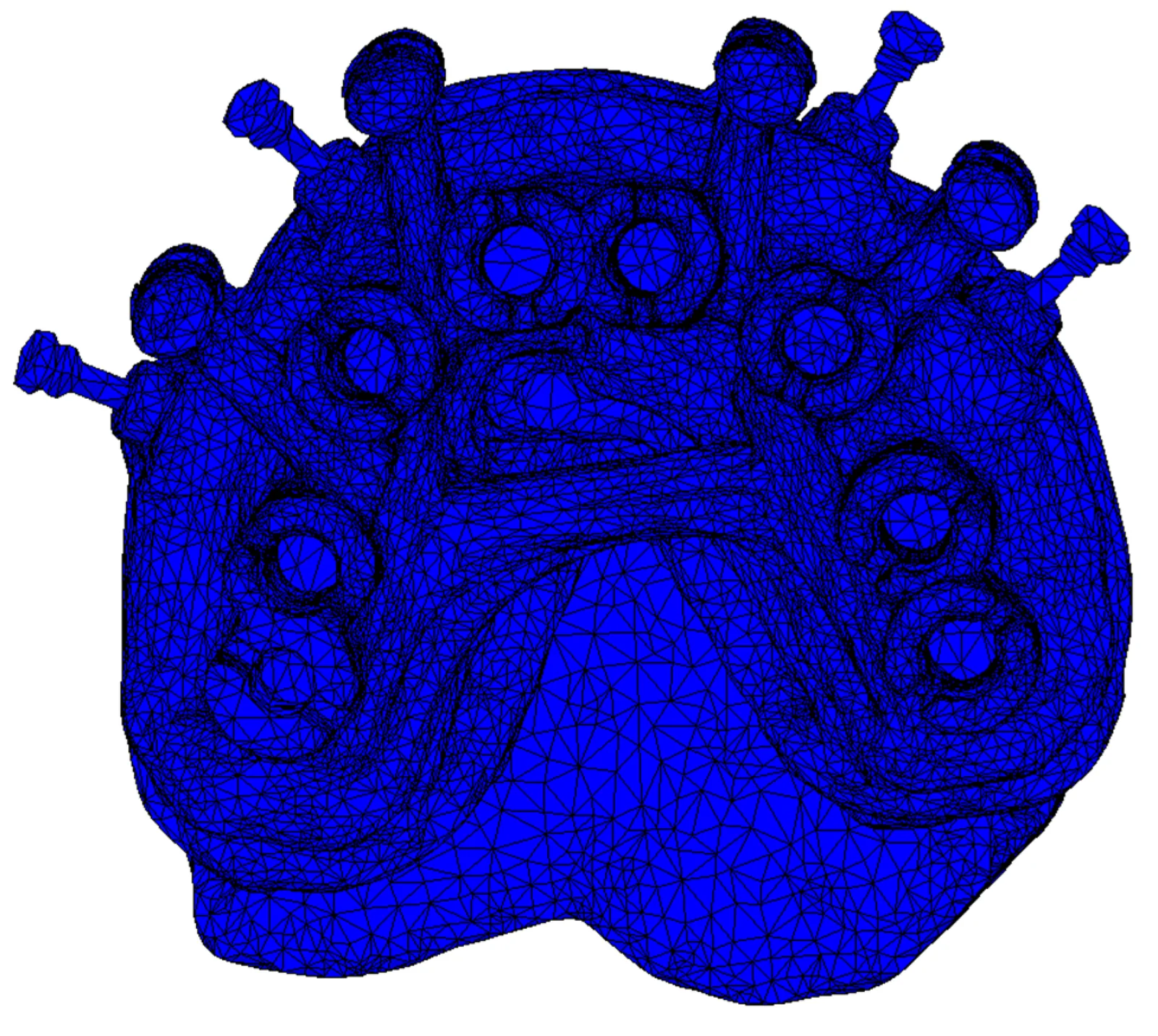
General representation of the whole studied system/assembly following the finite element analysis, showing correct components positioning and discretization.

**Figure 10:**
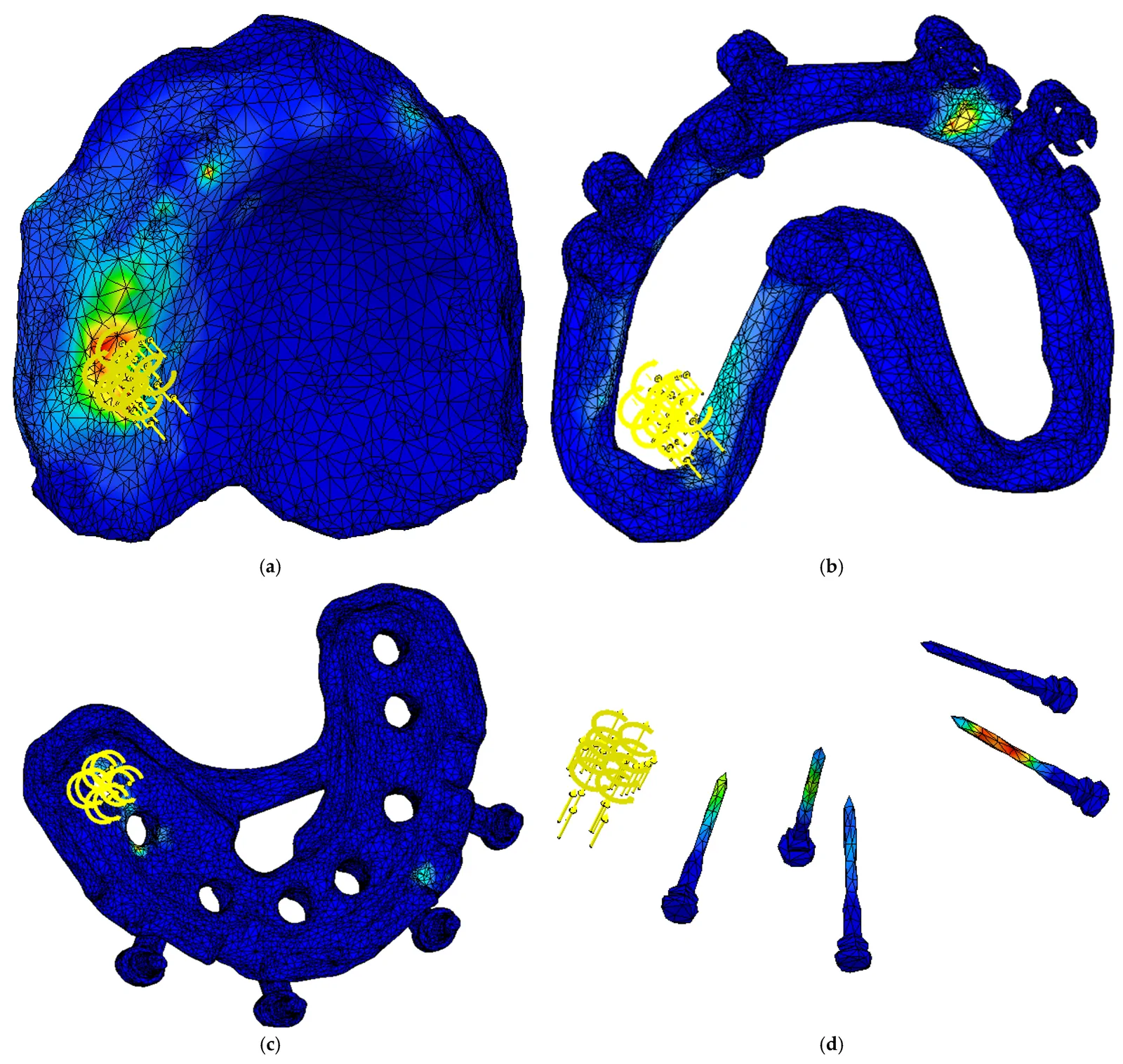
Von Mises stress distribution on the individual components of the analyzed system: (**a**) Maxilla component (maximum 2.34 MPa); (**b**) Base component (maximum 3.18 MPa); (**c**) Implants component (maximum 4.32 MPa); (**d**) Fixation Pin components (1.13–3.26 MPa). For all components, the maximum stress remained below the admissible strength of the assigned materials. Warm colors (red, orange, yellow) indicate regions of high stress, and cool colors (green, blue) indicate progressively lower stress.

**Figure 11:**
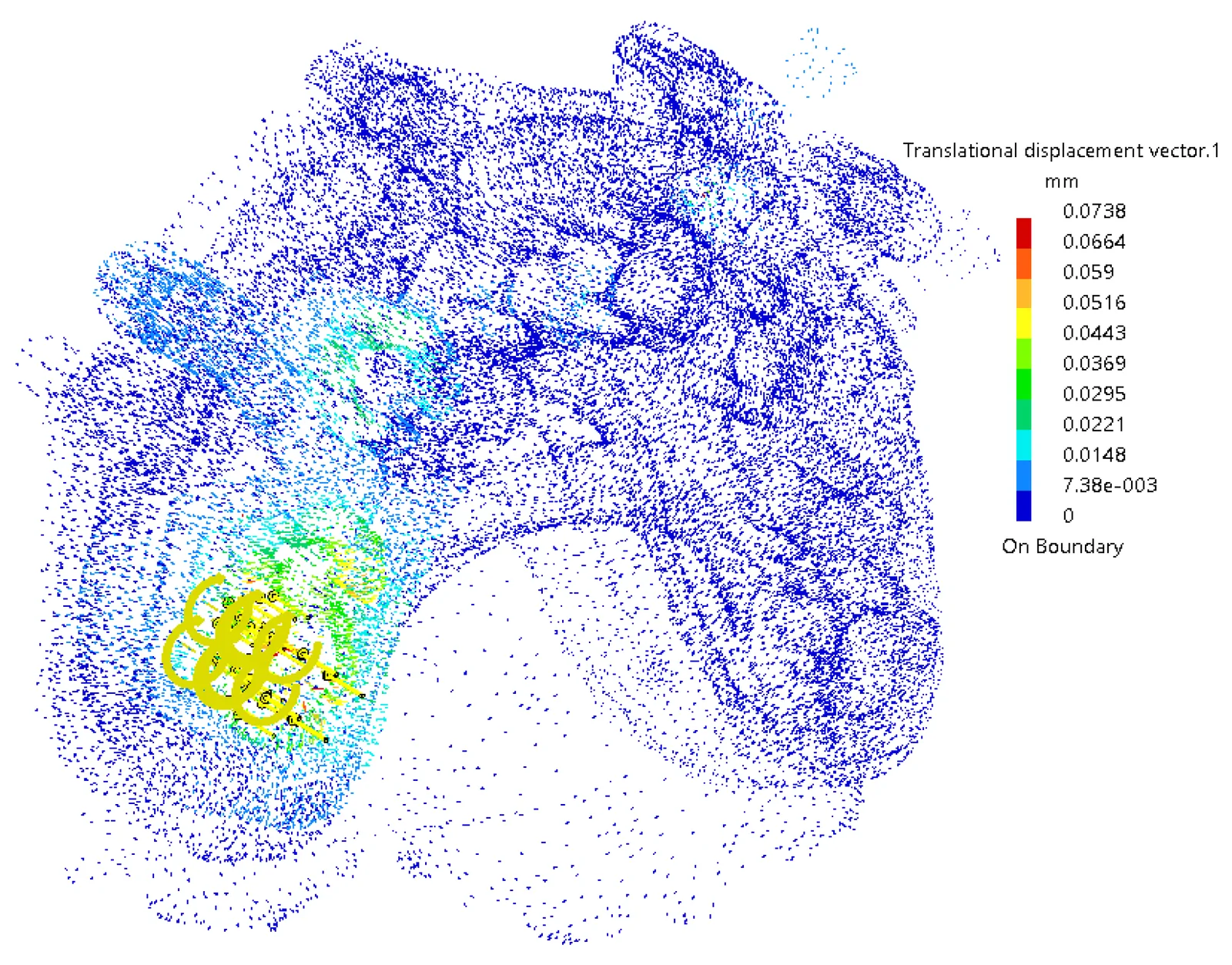
Deformation vectors of the components of the studied system; the highest values occur on the Implants guide (0.074 mm) and Base guide (0.026 mm) under Scenario 1.

**Figure 12:**
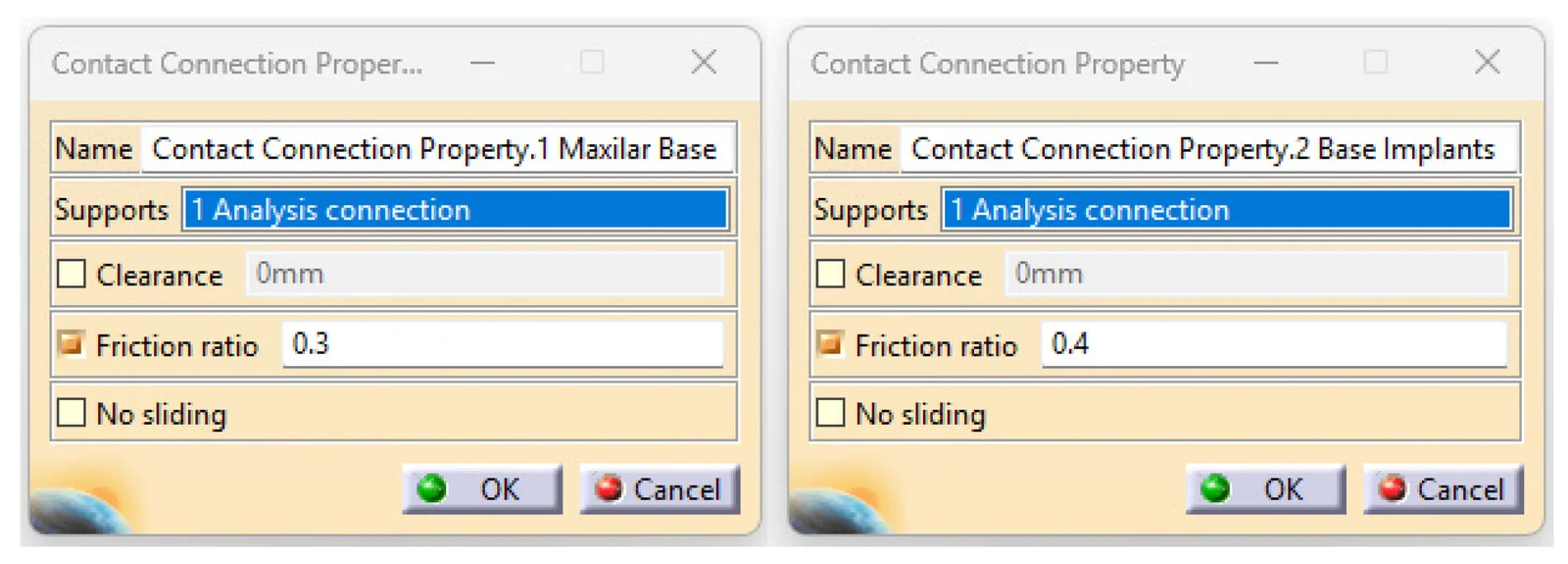
Examples of the frictional contact configuration implemented for Scenario 2 at the Maxilla–Base and Base–implant osteotomy guide interfaces.

**Figure 13:**
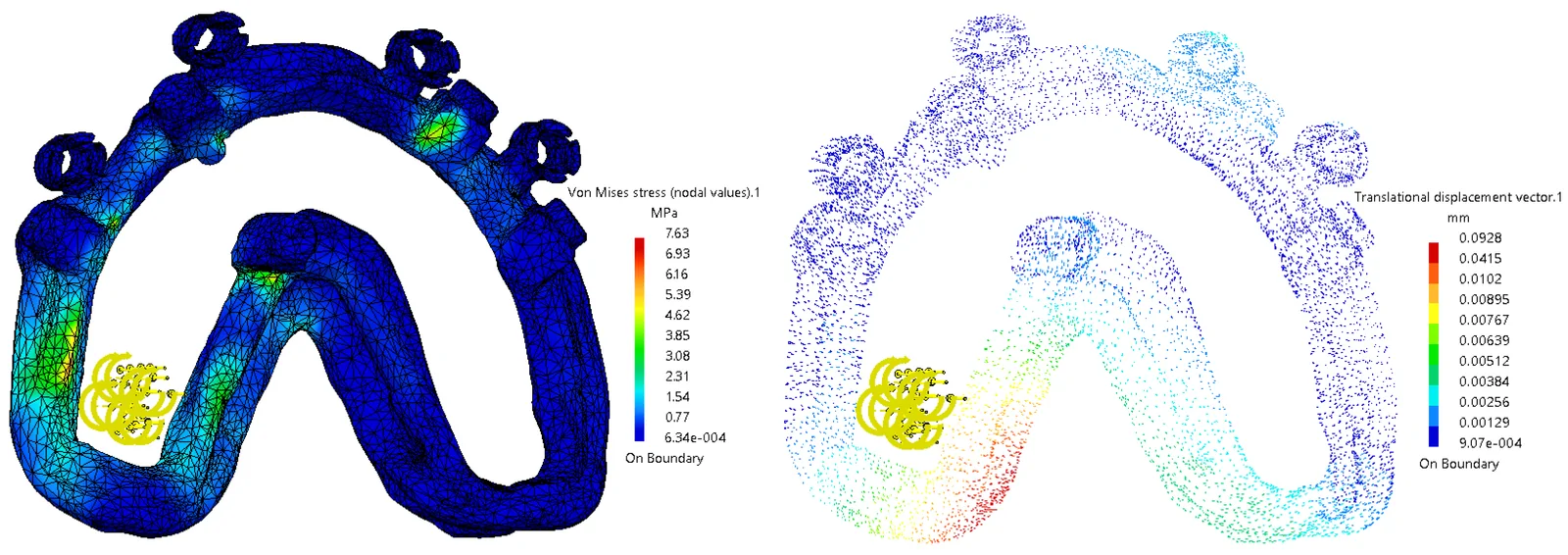
Stress and displacement vectors of the Base component under Scenario 2 (frictional contact).

**Table 1:** Material properties assigned to the components of the analyzed stackable guide system [11,12,13].

Component	Material	Young’s Modulus (MPa)	Poisson’s Ratio	Density (kg/m^3^)	Reference Strength (MPa)
Maxilla	Maxillary bone (homogenized)	13,000	0.3	1250	104
Base	E-Guide Resin	2600	0.3	1120	60
Implants	E-Guide Resin	2600	0.3	1120	60
Fixation pins	Ti-Grade-4 (Ti75A)	115,000	0.34	4500	550

**Table 2:** Summary of the maximum von Mises stresses, material-utilization ratios, and predicted deformations obtained under the two contact scenarios for each modeled component. Utilization is defined as the ratio of maximum stress to the corresponding reference strength.

Component	Material	Max. von Mises Stress (MPa) S1/S2	Reference Strength (MPa)	Utilization (%) S1/S2	Max. Deformation (mm) S1/S2
Maxilla	Maxillary bone (homogenized)	2.34/2.48	104	~2.25/2.38	0.00086/0.0011
Base	E-Guide Resin	3.18/7.63	60	5.3/12.7	0.026/0.093
Implants	E-Guide Resin	4.32/2.38	60	7.2/3.9	0.074/0.051
Fixation pins	Ti-Grade-4	1.13–3.26/1.11–3.94	550	<1.0	0.01/0.012

S1 = Scenario 1 (idealized no-sliding contact); S2= Scenario 2 (frictional contact).

## Data Availability

The original contributions presented in this study are included in the article. Further inquiries can be directed to the corresponding author.

## Abbreviations

The following abbreviations are used in this manuscript:

CBCT	Cone-beam computed tomography
FEA	Finite Element Analysis
FEM	Finite Element Method
STL	Standard tessellation language
DLP	Digital Light Processing
SLA	Stereolithography
HB	Brinell hardness
Base	Polymeric base guide (model component)
Implants	Implant osteotomy guide (model component)
Maxilla	Maxillary support component derived from the intraoral scan
PMMA	Poly(methyl methacrylate)
TEGDMA	Triethylene glycol dimethacrylate
UDMA	Urethane dimethacrylate
Bis-EMA	Bisphenol A ethoxylated dimethacrylate
Bis-GMA	Bisphenol A-glycidyl methacrylate
CATIA	Computer-Aided Three-dimensional Interactive Application
DICOM	Digital Imaging and Communications in Medicine
